# List of Upcoming Meetings

**DOI:** 10.1055/s-0038-1667027

**Published:** 2018-07-27

**June 2018**

1. Aortic Valve Repair Summit
  June 18-19, 2018
  Paris, France
Meeting information available at: http://www.eacts.org/educational-events/programme/2018avrsummit/

2. Critical Issues in Aortic Endografting 2018
  June 29-30, 2018
  Malmö, Sweden
Meeting information available at: http://critical-issues-congress.com

**July 2018**

3. CVC 2018: Arterial and Venous Endovascular Conference
  July 11-13, 2018
  Chicago, IL, USA
Meeting information available at: http://www.cvcpvd.com

**September 2018**

4. 6th International Meeting on Aortic Diseases
  September 12-14, 2018
  Liège, Belgium
Meeting information available at: http://www.chuliege-imaa.be/vpage.php

5. 3rd Barts Aortovascular Symposium 2018
  September 15-16, 2018
  London, United Kingdom
Meeting information available at: http://bartsaortovascularsymposium.org.uk/

6. European Society for Vascular Surgery, 32nd Annual Meeting
  September 25-28, 2018
  Valencia, Spain
Meeting information available at: http://www.esvs.org/valencia-2018/

